# Discussion at the Medico-Legal Society

**Published:** 1911-05-27

**Authors:** 


### DISCUSSION AT THE MEDICO-LEGAL SOCIETY.

At a meeting of the Medico-Legal Society held at 11 Chandos Street, W., on May 23 (Sir John Tweedy in the chair), Dr. J. Smith Whitaker, medical secretary to the British Medical Association, delivered an address on the National Insurance Bill. Having explained the objects and scope of the measure, he dealt with the position of medical men under the proposed scheme. He said that clause 14, in its present form, left it open to friendly societies and local health committees to make any kind of terms with medical practitioners. They could institute a system of capitation fees, fees per visit, or annual salaries, and they could leave it open for all medical practitioners in a district to attend insurance patients. Proceeding, he expressed the opinion that the intervention of the State between doctors and patients was regrettable but inevitable. If there must be this interference, it should not go further than was necessary. The present Bill, however, did carry this interference further than was necessary. Medical attendance would be provided for a great number of people who were capable of paying a private practitioner. He strongly urged the desirability of leaving the choice of the doctor with the patient, and he thought the arrangements for the administration of medical benefits should be in the hands of local committees and not friendly societies. In conclusion, he pleaded for unity of purpose and action among medical men in seeking to improve the Bill. In the course of the discussion which followed Dr. Smith Whitaker's address, Sir Henry Craik, M.P., expressed the opinion that the income limit should be £2 a week rather than £160 a year. He promised to do his best to place the views of the medical profession before the House of Commons.

