# A Correction

**Published:** 1911-06-03

**Authors:** 


### A Correction.

The fourth consideration in Mr. Harry Johnson's presentation last week of Sir Edward Wood's opinion as to the probable effect of the Insurance Bill on the Leicester Hospital Saturday Society should read: "Their [the members] sickness contributions under the Bill would *not* be greater, and would, in effect, be transferred from one account to another, and there was no reason to anticipate their withdrawal from membership." We much regret the accidental omission last week of the word now in italics.

